# Evolution of MicroRNA Genes in *Oryza sativa* and *Arabidopsis thaliana*: An Update of the Inverted Duplication Model

**DOI:** 10.1371/journal.pone.0028073

**Published:** 2011-12-14

**Authors:** Yun Zhang, Wen-kai Jiang, Li-zhi Gao

**Affiliations:** 1 Plant Germplasm and Genomics Center, Kunming Institute of Botany, The Chinese Academy of Sciences, Kunming, China; 2 Graduate School, Chinese Academy of Science, Beijing, China; University of Georgia, United States of America

## Abstract

The origin and evolution of microRNA (miRNA) genes, which are of significance in tuning and buffering gene expressions in a number of critical cellular processes, have long attracted evolutionary biologists. However, genome-wide perspectives on their origins, potential mechanisms of their *de novo* generation and subsequent evolution remain largely unsolved in flowering plants. Here, genome-wide analyses of *Oryza sativa* and *Arabidopsis thaliana* revealed apparently divergent patterns of miRNA gene origins. A large proportion of miRNA genes in *O. sativa* were TE-related and MITE-related miRNAs in particular, whereas the fraction of these miRNA genes much decreased in *A. thaliana*. Our results show that the majority of TE-related and pseudogene-related miRNA genes have originated through inverted duplication instead of segmental or tandem duplication events. Based on the presented findings, we hypothesize and illustrate the four likely molecular mechanisms to *de novo* generate novel miRNA genes from TEs and pseudogenes. Our rice genome analysis demonstrates that non-MITEs and MITEs mediated inverted duplications have played different roles in *de novo* generating miRNA genes. It is confirmed that the previously proposed inverted duplication model may give explanations for non-MITEs mediated duplication events. However, many other miRNA genes, known from the earlier proposed model, were rather arisen from MITE transpositions into target genes to yield binding sites. We further investigated evolutionary processes spawned from *de novo* generated to maturely-formed miRNA genes and their regulatory systems. We found that miRNAs increase the tunability of some gene regulatory systems with low gene copy numbers. The results also suggest that gene balance effects may have largely contributed to the evolution of miRNA regulatory systems.

## Introduction

Small RNA molecules of about 20–30 nucleotides have emerged as significant regulators of gene expression and genome stability [1]. In plants, microRNAs (miRNAs) are one of two distinct types of small RNAs that are currently understood. The short interfering RNAs (siRNAs) are typically processed by Dicer proteins from long, perfectly double-stranded RNA (dsRNA) precursors. miRNAs are produced by Dicer-catalyzed excision from hairpin structure precursors that are transcribed from miRNA genes [2], [3], [4]. The majority of miRNAs are able to negatively regulate gene expression via direct RNA-induced silencing complex (RISC) binding to target mRNAs, resulting in transcript degradation or translational repression, while small fractions have developed specific properties that regulate other transcriptional or posttranscriptional silencing pathways [5]. Increasing references have shown that miRNAs function as key regulators of nearly all essential cellular processes, particularly in the development and stress responses [6]. With a better knowledge of functions of miRNAs, we could easily speculate on their potential applications, such as designing desired crops with better yields [7], increased resistance to disease [8], and the adaptation to environmental extremes [9]. In addition to answering fundamental biological questions regarding the role of miRNAs, however, a comprehensive understanding of the origin and evolution of miRNAs is intriguing but largely unsettled.

In recent years, great efforts have been devoted towards the investigation of the origin of miRNA genes and consequently several modes have been proposed. In plants, for example, based on the observation that some miRNA precursors had extended similarity beyond miRNA sequences with target genes, Allen and colleagues [10] proposed the inverted duplication model. Under the hypothesis, these miRNA genes were supposedly generated from inverted duplication events of one of their target genes by forming two adjacent gene segments in either convergent or divergent orientation. Subsequent discovery that a large proportion of miRNA genes laying within transposable elements (TEs) or pseudogenes, made scientists believe that these miRNA genes might arise from TEs [11], [12], [13], [14] or pseudogenes [15], [16], [17] by some types of mechanisms which, thus far, have not been well known. Genome-wide analysis of miRNA genes in *Arabidopsis thaliana* further made known that many of them arose from the whole genome duplications (WGDs), tandem duplications and segmental duplications followed by their dispersal and diversification, somewhat similar to the behavior of protein-coding genes [18]. Moreover, an extensive investigation of young miRNA genes in *A. thaliana* showed that their origin could not be fully explained by the above-mentioned hypothesis, and thus these researchers even proposed that some miRNA genes might have been derived from random sequences and spontaneously formed from foldback sequences [19].

To date, the origin of miRNA genes has been limitedly investigated under one or another model organism alone by using a subset of them. Even motivated by recent advances in the whole genome sequencing as well as deep sequencing of small RNA libraries, a genome-wide picture of how miRNA genes originate is still too far away to be outlined for any organism, to say nothing of profound insights into how miRNA genes of different origins diversify. While hypotheses for *de novo* generation of miRNA genes have been proposed, by and large, little has been explored, to any great depth, regarding mechanisms of their origins. As for either the inverted duplication model or the hypothesis of “miRNA genes derived from TEs or pseudogenes”, for instance, we are unable to elucidate the mystery of the origin and particularly precise mechanisms behind, let alone the discrepancy and relationships between different modes of origin and those *de novo* origin mechanisms in particular. Undoubtedly, a comprehensive understanding towards evolutionary history of *de novo* generated miRNA genes and their regulatory systems remains to be understood.

In this study, we performed a genome-wide characterization of miRNA genes in two well-annotated model plants, *Oryza sativa* and *A. thaliana*, and comprehensively investigated and compared modes of miRNA gene origins (Figure S1). Differences and relationships among these modes were further examined for these two genomes. To better illustrate how miRNA gene *de novo* generated, we compared miRNA genes with binding target sequences between two closely related genomes, *O. sativa* ssp. *japonica* and *O. sativa* ssp. *indica*. Lastly, we explored the evolution of miRNA genes and their regulatory systems by distinguishing different features between *de novo* generated and conserved miRNA genes and their regulatory systems. Taken them together, attempts were made to elucidate how miRNA gene originate and evolve towards a genome-scale perspective in flowering plants.

## Results

### A landscape of miRNA gene origins in *O. sativa* and *A. thaliana*

**Figure 1:**
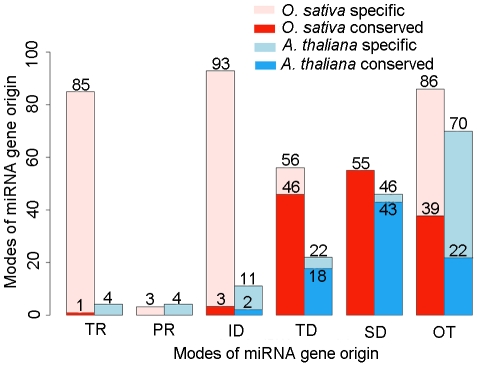
Quantitative contributions of different miRNA gene origin modes in *O. sativa* and *A. thaliana* genomes. Horizontal axis indicates origin modes of miRNA genes, TR represents TE-related, PR represents pseudogene-related, ID represents inverted duplication, TD represents tandem duplication, SD represents segmental duplication, and OT represents other miRNA genes could not fit any above-mentioned categories. Vertical axis indicates miRNA gene number. Mistyrose bars represent *O. sativa* specific miRNA genes. Red bars represent conserved miRNA genes (both present in *O. sativa* and *A. thaliana*) in *O. sativa* genome. Lightblue bars represent *A. thaliana* specific miRNA genes. Blue bars represent conserved miRNA genes (both present in *O. sativa* and *A. thaliana*) in *A. thaliana* genome.

According to their modes of origin, we classified the whole dataset of miRNA genes identified in the *O. sativa* and *A. thaliana* genomes into six subsets: TE-related, pseudogene-related, inverted duplication-generated, tandem duplication-generated, segment duplication-generated, and others. Here, TE- and pseudogene-related miRNA genes referred to the subsets with their precursor sequences overlapped or joined with TEs or pseudogenes. These miRNA genes, as previously reported, might originate from TEs or pseudogenes. Inverted duplication-generated miRNA genes were highly similar to target genes and they might arise from inverted duplication events by not well-known mechanisms. Tandem duplication-generated and segmental duplication-generated miRNA genes came up from gene duplication events with a mode same as protein-coding genes. If the identified miRNA genes could not fit any above-mentioned categories, then they fell into the last subset. (Figure. 1, Table S1). In both plant species, most of the TE-related, pseudogene-related and inverted duplication-generated miRNA genes were species-specific. Of the 290 identified miRNA genes in the *O. sativa* genome, a large number was able to account for TE-related (85, 29.3%) and inverted duplication-generated (93, 32.1%), respectively. In comparison, the proportions of miRNA genes both originated from TEs (4/142, 2.8%) and generated through inverted duplication events (11/142, 7.7%) in *A. thaliana* were significantly lower than those in *O. sativa* (Chi-Square tests, *P*<10^−3^).

By using RepeatMasker program, we found that TE content in *O. sativa* (38.39%) was at least two-fold higher than in *A. thaliana* (15.82%). Of total sequences of 43,137 and 24,016 bp miRNA genes in *O. sativa* and *A. thaliana*, therefore, 16,560 and 3,799 bp should overlap with TEs in a random state. In fact, however, overlapped sequences were 16,048 bp (96.91%) and 612 bp (16.11%) in *O. sativa* and *A. thaliana*, respectively, indicating that TE content overlapped with miRNA genes in *O. sativa* was significantly larger than in *A. thaliana* (Chi-Square test, *P*<10^−3^). Furthermore, TEs related to miRNA genes and their binding sites were mainly miniature inverted-repeat transposable elements (MITEs) in *O. sativa*. Of TEs related to miRNA genes and their targets in the *O. sativa* genome, DNA/Stowaways accounted for approximately 67.34% and 82.43%, respectively. These proportions were much higher than the fraction of DNA/Stowaways on average, comprising 17.33% of the detected TEs in the *O. sativa* genome on the whole (Chi-Squere tests, *P*<10^−3^). In *A. thaliana*, however, TEs related to miRNA genes and their targets were predominantly other TEs, such as DNA/MuDR, DNA/hAT, LTR/Gypsy and RC/Helitron (Table S2). Divergence e-values of TEs, which were estimated on the basis of sequence divergence between the TE sequences and family consensus sequences (see Table S2), further showed that average ages of TEs related to miRNA genes in *O. sativa* (divergence e-value = 15.13) was older than those related to miRNA targets (divergence e-value = 12.86, t-test *P*<10^−3^). Nevertheless, average age of TEs (divergence e-value = 13.40) related to miRNA genes was younger than those related to miRNA targets (divergence e-value = 23.20, t-test *P* = 0.062) in *A. thaliana*. We found that miRNA genes generated from different origin modes possessed diverse features, evidenced by their function, sequence, structure, distribution, expression, regulation, conservation and polymorphisms (Table S3).

### The majority of TE- and pseudogene-related miRNA genes have originated through inverted duplication events

**Figure 2:**
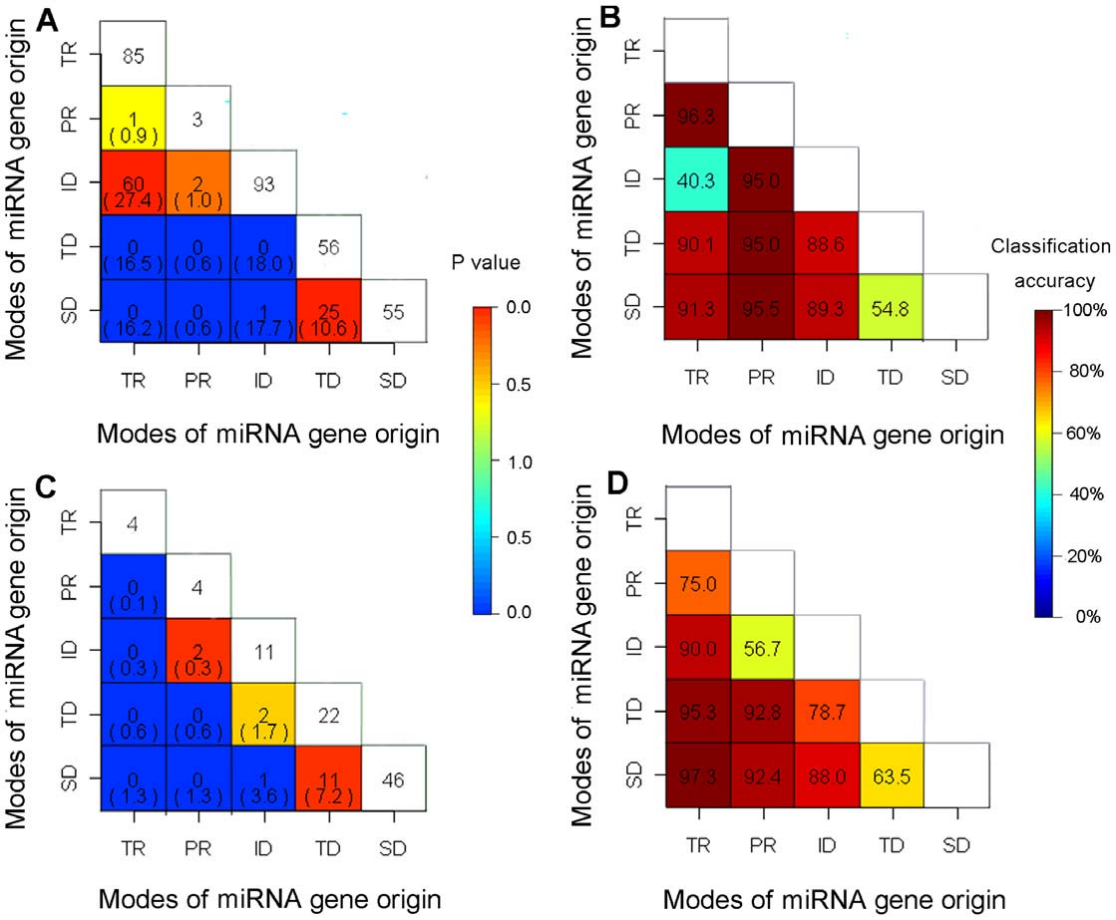
Relationships among different origin modes of miRNA genes. (A) and (B) are for *O. sativa*, while (C) and (D) are for *A. thaliana*. TR represents TE-related, PR represents pseudogene-related, ID represents inverted duplication, TD represents tandem duplication, and SD represents segmental duplication. (A) and (C) are intersection number of miRNA gene sets with different origin modes. Number in the center of each lattice represents intersection number of miRNA gene sets with different origin modes represented by vertical and horizontal axes, and number in parentheses is values of random state deduced from 100,000 Monte Carlo simulations. Lattice color represents P-value between real and simulation values, red lattice represents that real value is larger than simulation value, and blue lattice represents that real value is smaller than simulation value. (B) and (D) are classification accuracy of miRNA gene sets with different origin modes.

In this study, two approaches were used to better illustrate relationships among different modes of miRNA gene origins. We first attempted to ensure whether intersection number of these datasets was smaller or larger than the status at random. For this purpose, we performed Monte Carlo simulations to estimate intersection number at random. As shown in Figure 2A and 2C, in *O. sativa*, intersection number of TE-related and inverted duplication was much larger than the status at random (*P*<10^−4^). In *A. thaliana*, intersection number of pseudogene-related and inverted duplication was larger than the status at random (*P* = 0.0301). Furthermore, in both plant species, intersection number of TE-related and tandem duplication, TE-related and segmental duplication, pseudogene-related and tandem duplication, pseudogene-related and segmental duplication was much smaller than the status at random (*P*<10^−4^).

We next came to confirm whether miRNA gene features of these datasets, as described in Table S3, were similar to or different from each other for both *O. sativa* and *A. thaliana*. Here, approaches of pattern recognition were applied to test the similarity of miRNA genes with respect to modes of their origin across different datasets. If the classification accuracy between two datasets is relatively high, one might expect that similarities of miRNA gene features of these two datasets are relatively low, and *vice versa*. As shown in Figure 2B and 2D, similarities of miRNA gene features between TE-related and inverted duplication in *O. sativa*, as well as pseudogene-related and inverted duplication in *A. thaliana*, were relatively high. However, similarities of miRNA gene features between TE-related and tandem duplication, TE-related and segmental duplication, pseudogene-related and tandem duplication, as well as pseudogene-related and segmental duplication, were relatively low. We further analyzed the other two types of miRNA genes as below: first, miRNA genes with its target sites overlapped with TEs; and second, TE-related miRNA genes with its target sites overlapped with the same TEs. It is our findings that Monte Carlo simulation and pattern recognition of these miRNA genes were consistent with the above-analyzed TE-related miRNA genes (Table S4).

Overall, the above-mentioned results together support that the majority of TE-related and pseudogene-related miRNA genes have originated through inverted duplication instead of segmental or tandem duplication events. Additionally, the proportion of TE induced inverted duplication events in *O. sativa* was higher than in *A. thaliana*. Such findings also indicate that inverted duplication may not be correlated to segmental or tandem duplication. Nonetheless, it deserves to mention that tandem duplication was associated with segmental duplication (Figure 2).

### Origins of *de novo* generated miRNA genes

To elucidate molecular mechanisms of how TE-derived miRNA genes were *de novo* generated, we focused on miRNA genes whose sequences and at least one target site entirely overlapped with the same TEs, and these target sites fitted the inverted duplication model as well. Of the 85 miRNA genes related to TEs in the *O. sativa* genome, 45 met the above-described criteria (Table S5). Among the four miRNA genes related to TEs in the *A. thaliana* genome, however, none of them satisfied the above-described requirements. Thus, we took a total of 45 candidate miRNA genes identified in the *O. sativa* genome into further consideration. Of them, six overlapped with non-MITEs, while the other 39 were partially covered by MITEs. It is attractive to find that, of miRNA genes overlapped with non-MITEs, four (4/6, 66.67%) were related to two copies of inverted non-MITEs with >80% sequence similarity. Whereas, among miRNA genes related to MITEs, there were only one (1/39, 2.56%) associated with pairs of inverted MITEs with >80% sequence similarity (Table S5). Statistical analysis showed that they were significantly different (Chi-Square test, *P*<10^−3^). We then compared features of non-MITE-related miRNA genes with MITE-related ones and found that some of them were different (Table S6). For example, the family size and target number of non-MITE-related miRNA genes were much smaller than MITE-related ones. Approaches of pattern recognition also indicate that these two types of miRNA genes exhibited moderately high difference (classification accuracy was 82.2%). These results thus suggest that mechanisms of miRNA gene *de novo* generated from non-MITEs and MITEs may be different from one to another.

**Figure 3:**
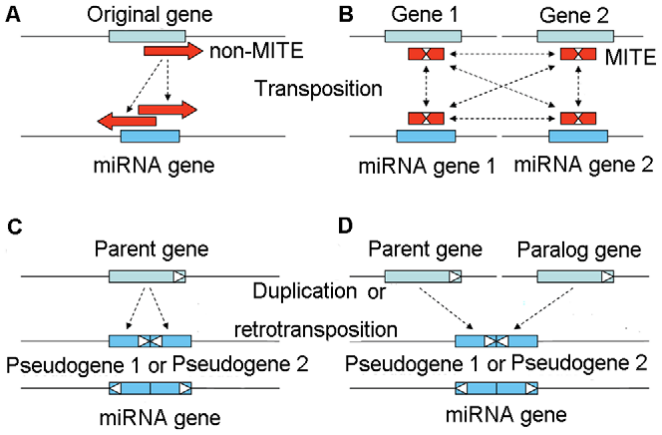
Schematic illustrations of the proposed *de novo* origin mechanisms of miRNA genes. (A) Mechanism of miRNA gene *de novo* originated from non-MITE transposable element. (B) Mechanism of miRNA gene *de novo* originated from MITE. (C) Mechanism of miRNA gene *de novo* originated from pseudogenes arose from one parent gene. (D) Mechanism of miRNA gene *do novo* originated from pseudogenes arose from one parent gene and its paralogous gene.

Our results showed that the inverted duplication model [10] may better explain non-MITEs mediated duplication due to long non-MITEs in length. As shown in Figure 3A, two identical or closely related copies of non-MITEs, which originated from target gene, inserted into adjacent regions in opposite orientation and formed miRNA gene. As for MITEs mediated “inverted duplication”, however, it is clear that MITE-related miRNA genes possessed larger number of targets than non-MITE related ones (Table S6) and those MITEs related to miRNA targets appeared relatively young (Table S2). Given the characteristics of MITEs, such as palindromic structures and small size, we wonder whether MITEs could be transposed from miRNA genes to target genes or any other directions with the exception from target genes to miRNA genes.

For MITEs that overlapped with these 39 miRNA genes and their targets, we collected upstream and downstream flanking sequences with a series of length cutoffs of 100 bp, 300 bp and 500 bp, respectively, and then combined two sides together. We found that the cutoff of 500 bp appeared sufficiently strict and thus was suitable for further sequence comparisons. Then, we blasted them against the genome sequences of *93-11* [20], a cultivar of *O. sativa* ssp. *indica*. There were two MITEs, which jointed flanking sequences of 1000 bp and orthologous regions in the *93-11* genome, were almost identical with BLASTN searches (E-value: 0.0 and 0.0, respectively; similarity: 99.8% and 97.11%, respectively). The first MITE was overlapped with MIR806h (Figure S2), while the second one was associated with the target of MIR446 on LOC_Os08g05620 (Figure S3). Then, phylogenetic trees were reconstructed by using all of the same MITEs overlapped with miRNA genes, miRNA targets and other miRNA family members for each of these two miRNAs as well as consensus sequences of two MITE families. Consensus sequences are often used as a reasonable approximation of ancestral sequences. Phylogenetic analyses in this study show that the distances between the MITE sequences which overlapped with MIR806h and its consensus sequence were relatively large. The finding suggests that the MITE sequences which overlapped with MIR806h were relatively young (Figure S4A). We similarly observed relatively large distances between the MITE sequences which overlapped with target of MIR446 on LOC_Os08g05620 and its consensus sequence, indicating that this MITE sequence might also be comparatively young (Figure S4B). Thus, it could be assumed that, in addition to target gene to miRNA gene, the direction of MITE mediated “inverted duplication” may initiate from miRNA gene to target gene or even other directions (Figure 3B). Since an outgroup genome has not become available, it is unlikely to certainly determine whether these MITEs inserted into or became lost from the genome. Regardless of the gain or loss, MITEs-mediated miRNA gene or miRNA target site generation should play an important role in *de novo* generation of miRNA genes as well as their targets.

To further clarify molecular mechanisms of how pseudogene derived miRNA gene was *de novo* generated, we investigated two miRNA genes in *O. sativa* and two miRNA genes in *A. thaliana* which overlapped with pseudogenes and fitted inverted duplication model (Figure 2). Of them, we identified one miRNA gene in *A. thaliana*, which appeared overlapped with two adjacent inverted paralogous pseudogenes. The parent gene of these pseudogenes acted as binding target of this miRNA (Table S7). This miRNA might originate from two adjacent pseudogenes with identical or similar sequences derived from the same target gene in opposite orientation (Figure 3C). Intriguingly, two miRNA genes in *A. thaliana* overlapped with two different pseudogenes and their parent genes were paralogous (Table S8). We therefore hypothesize that few miRNA genes might even originate through the juxtaposition of two physically closed pseudogenes from a gene family (Figure 3D).

### Different features between *de novo* generated and conserved miRNA genes and regulatory systems

We came to compare features of *de novo* generated and conserved miRNA genes. Here, *de novo* generated miRNA genes included TE-related, pseudogene-related and inverted duplication generated miRNA genes. Whereas, conserved miRNA genes consisted of tandem duplication and segmental duplication generated miRNAs. We found significant differences between these two types of miRNA genes concerning their function (Table S3), sequence, structure, distribution, regulation, expression and evolution (Table 1).

**Table 1:** Differences between *de novo* generated and conserved miRNA genes in *O. sativa* and *A. thaliana*.

	*O. sativa*			*A. thaliana*		
	*de novo*	Conserved	*P*-value^a^	*de novo*	Conserved	*P*-value
Adenine at first nucleotide	39.29%	5.13%	*<*10^−3^	13.04%	5.48%	*<*10^−3^
miRNA length (nt)	22.37	21.00	*<*10^−3^	21.74	20.91	*<*10^−3^
Precursor length (nt)	171.85	119.17	*<*10^−3^	196.32	140.01	0.001
Stem length (nt)	83.95	57.42	*<*10^−3^	96.71	68.04	0.001
Distance between miR and loop(nt)	43.27	22.78	*<*10^−3^	41.16	22.10	0.001
Loop length (nt)	5.95	6.33	0.176	4.90	5.93	0.113
Match ratio of hairpin^b^	80.03%	74.34	*<*10^−3^	75.52%	72.85%	0.097
siRNA (#)^c^	1.50	0.40	*<*10^−3^	4.11	1.38	*<*10^−3^
Multi-miRNA^d^	6.08%	0.00%	0.008	21.05%	0.00%	*<*10^−3^
Location^e^	34.81%	7.76%	*<*10^−3^	15.79%	6.94%	0.266
Promoter (#)^f^	0.52	1.29	/	0.58	1.35	/
SSRs (#)^g^	0.46	0.95	*<*10^−3^	0.32	0.72	0.020
Target number (#)	20.69	8.53	*<*10^−3^	10.90	8.25	0.244
Target gene expression intensity^h^	435.20	562.71	0.005	5.80	6.75	0.005
Base pairings of cleaving site^i^	1.63	1.80	*<*10^−3^	1.56	1.82	*<*10^−3^
Family size (#)	5.53	13.46	*<*10^−3^	1.53	6.12	*<*10^−3^
Conservation^j^	1.23	8.70	*<*10^−3^	1.58	8.04	*<*10^−3^
miRNA expression intensity^k^	/	/	/	7.58	41.68	0.106
Polymorphic site density of pre-miRNA^l^	/	/	/	4.41%	1.50%	0.002
Polymorphic site density of miRNA	/	/	/	0.35%	0.23%	0.715
Polymorphic site density of target	/	/	/	4.44%	2.46%	0.118
Methylcytosine density^m^	/	/	/	2.74%	0.09%	*<*10^−3^
Promotor methylcytosine density^n^	/	/	/	1.08%	0.09%	*<*10^−3^

a t-test.

b Number of pairing bases/hairpin length.

c Number of siRNAs encoded by miRNA precursor.

d Percentage of miRNA precursor encoding more than one miRNA.

e Percentage of miRNA genes located in coding gene regions.

f Average promoter (TATA box) and enhancer number of a miRNA gene.

g Simple sequence repeats number adjacent to miRNA gene within 1 kb region.

h Mean of all gene expression intensity is 403.67 and 6.14 in *O. sativa* and *A. thaliana* respectively.

i Watson-Crick pairing number of miRNA cleaving site (10 and 11 nucleotides) on miRNA/target duplex.

j Orthologous number of miRNA gene in 14 plant species in miRbase.

k miRNA expression data, polymorphism site data and DNA methylation data only have *A. thaliana* data.

l Pre-miRNA polymorphic site number/pre-miRNA length. Polymorphic site density of the whole genome is 6.66%.

m Pre-miRNA DNA methylation site number/pre-miRNA length. Methylcytosine density of the whole genome is 0.98%.

n 1 kb upstream of pre-miRNA DNA methylation site number/1000.

In order to understand the evolution and interaction of miRNAs and their target genes which form the whole co-evolution system, as shown in Table 2, features of *de novo* generated and conserved miRNA regulatory systems were characterized. It is of interest to find that, within such a regulatory system, *de novo* generated miRNAs failed to make statistical difference in comparisons with overall gene set in the genome. However, there was statistical difference in the conserved miRNA regulatory system. Target gene copy number of conserved miRNA genes, for example, was lower than average level of the entire set of genes; target gene alternative splice number of conserved miRNA genes was higher than average level of overall genes; and the percentage of target genes in segmental duplication blocks of conserved miRNA genes was larger than overall set of genes. However, we observed that the percentage of target genes in tandem duplication arrays of conserved miRNA genes did not show obvious difference, or lower than overall genes.

**Table 2:** Features of miRNA regulatory system in *O. sativa* and *A. thaliana*.

	*O. sativa*				*A. thaliana*			
	*de novo* a	Conserved^b^	All genes	*P*-value^c^	*de novo*	Conserved	All genes	*P*-value
Copy number^d^(#)	4.31	3.64	4.67	*<*10^−3^	11.84	3.82	6.08	*<*10^−3^
Splicing number^e^ (#)	1.77	1.48	1.27	*<*10^−3^	1.21	1.43	1.17	*<*10^−3^
Segmental duplication^f^	27.96%	39.55%	22.13%	*<*10^−3^	84.26%	89.86%	65.00%	*<*10^−3^
Tandem duplication^g^	12.01%	12.63%	11.33%	0.313	31.36%	9.38%	13.86%	0.004

a Target genes of *de novo* generated miRNA genes.

b Target genes of conserved miRNA genes.

c t-test between target genes of conserved miRNA genes and all genes.

d Average level of all (target) genes.

e Average level of all (target) genes.

f Percentage of (target) genes in segmental duplication blocks.

g Percentage of (target) genes in tandem duplication arrays.

## Discussion

### Genome-wide patterns of miRNA gene origins in *O. sativa* and *A. thaliana*

The origins of miRNA genes have attracted extensive attention in recent years, and a variety of hypotheses have been continuously proposed [10], [11], [12], [13], [14], [15], [16], [17]. Nevertheless, the question remains unanswered at a genome-wide level. In this study, we systematically investigated modes of miRNA gene origins and observed different patterns in two well-annotated *O. sativa* and *A. thaliana* genomes. A large proportion of miRNA genes in *O. sativa* were TE- and MITE-related miRNAs in particular, whereas the fraction of these miRNA genes much decreased in *A. thaliana*. One may wonder what have resulted in these differences between *O. sativa* and *A. thaliana* genomes. The most possible explanation is that, as shown in Table S2, TE content in *O. sativa* (38.39%) was at least two-fold higher than in *A. thaliana* (15.82%), leading to that TE composition overlapped with miRNA genes in *O. sativa* was significantly larger than in *A. thaliana*. In addition, as a modern monocotyledonous plant, *O. sativa* undergoes both natural and artificial selections and might encompass remarkably high rate of genomic changes, such as rapid nucleotide substitutions [21], abundance in ongoing individual gene duplications [22], high rates of gene and intron loss [23], and massive TE insertions, particularly MITE insertions into gene-rich genomic regions [9], [24]. These TE insertions have unquestionably provided source materials for the assembly and tinking of gene regulatory systems, in which massive TE-derived miRNA genes as well as miRNA targets were involved. Besides, the enhanced rate of molecular changes endows these organisms with increased evolvability. But if the evolvability is much higher than its robustness, such a gene regulatory system might collapse due to detrimental mutations. Within these systems, it is possible that the abundance in miRNAs, particularly novel miRNAs and elaborate regulation systems of miRNAs, such as four Argonaute (AGO) 1 homologs encoded in *O. sativa* but only one in *A. thaliana* [25], might bring balance to such a rapidly evolved system by providing regulatory robustness to a certain extent. Regulatory robustness might indirectly provide genetic robustness which allows genetic variation to accumulate in a cryptic state. Thus, the system possesses a store of concealed, potential genetic variability known as “evolutionary capacitors” [26], and then, the “evolutionary capacitors” increased sustainable evolvability of the system.

### Modes of miRNA gene origins

Potential mechanisms of how miRNA genes have been *de novo* generated lies at the core of understanding the origin of miRNA genes. By means of genome-wide analysis of miRNA genes in *A. thaliana*, Allen and colleagues first put forward the inverted duplication hypothesis, proposing that these miRNA genes were generated from inverted duplication events of one of their target genes [10]. Our study further confirmed that, in both *A. thaliana* and *O. sativa*, it was inverted duplication instead of segmental or tandem duplication events that mainly gave rise to the origin of TE-related and pseudogene-related miRNA genes. Based on the above-described data, we hypothesize the four possible mechanisms, which may be used to explain *de novo* generated novel miRNA genes from TEs and pseudogenes in flowering plants (Figure 3). It is notable that miRNA genes generated by each of these mechanisms were capable of being detected under the inverted duplication model. Nevertheless, these miRNA genes of diverse origins may have experienced different paths of molecular mechanisms, such as MITE-induced mechanism in particular, by means of which miRNA targets could insert into target genes through MITE transposition events.

We next question why TEs and pseudogenes are mostly able to serve as source materials to derive miRNA genes instead of other compositions within a genome. Recent studies suggest that small RNAs as guardians of gene regulatory system may come out very early even before the emergence of DNA world to protect primitive gene regulatory system against the instability [27], [28]. The first biological function attributed to small RNAs may be the defense against transposons and viruses [29], and thus miRNA regulatory system appears rather young [30]. It is possible that endogenous and exogenous impacts on the stability of gene regulatory system became enhanced with the gradually increased complexity of organisms. Mutations may have impacted on gene regulatory system during evolutionary processes. Stochastic fluctuation often occurs during progressively complicated developmental processes, in which gene expression program undergoes dramatic altering. Moreover, various perturbations may also come out under environmental stresses. Some parts of primitive small RNA regulatory system may co-opt as regulators of genes instead of transposons and viruses to provide the robustness for gene regulatory system. On one hand, to provide genetic robustness during evolutionary processes, siRNA regulatory system could partly repress and even eliminate redundant gene copies [31], such as homologous transgene [32], [33], polyploidy [34] and paramutation [35]; on the other hand, various elaborate mechanisms of miRNA regulatory systems [36], [37], [38], [39], [40] might also quickly evolve to provide the robustness during developmental processes and environmental turmoil. These lately evolved systems almost, if not all, have originated from primitive small RNA defense system, and thus they may take advantage of some cellular mechanisms that already exist in the primitive system, such as the uses of TEs as source of small RNAs.

Pseudogenes are usually classified into two major categories, named as processed pseudogenes and duplicated pseudogenes [17]. The behavior of processed pseudogenes, which is quite similar to retrotransposons, has made them become an alternative sources of miRNA genes [41]. Pseudogenes generated from gene duplications are redundant copies, and few of them may serve as ultimate sources of miRNA genes. Therefore, it is still possible that some small RNA pathways repressing redundant gene copies may also be capable of generating miRNA due to the exaptation.

There is a great challenge ahead insofar that we were unsuccessful in identifying their origin modes of small portions of miRNA genes. We do believe that plenty of them could originate from one of the above-described paths. However, with the evolution of plant genomes, the degeneration of their footprints has already made difficulties in accurately detecting their origin modes. It is also probably true that there exist unknown mechanisms of origin [19].

### Evolutionary processes of miRNA genes and regulatory systems

Our findings show that *de novo* generated miRNA genes have made remarkable differences from conserved ones (Table 1). These differences may be a sign of evolutionary processes from *de novo* generated to conserved miRNA genes, such as decreased precursor length, enriched regulatory elements, more canonical processing and increased expression intensity [42], [43], [44], [45].

It is of great interest to discover that newly generated miRNA regulatory systems failed to show obvious characteristics; however, with the evolution of these systems, they progressively obtain significant features. Our results suggest that, target gene copy number of conserved miRNA genes was lower than average level estimated from the whole set of genes, while target gene alternative splice number of conserved miRNA genes was higher than average levels throughout the genome. The findings are consistent to recent studies, which reported that these genes with low-copy number have relatively more enriched alternative splicing number as well as antisense transcript regulation [46], [47], [48]. Furthermore, MITEs tend to insert into genes with low-copy number [24], and pseudogenes also preferentially come from such genes [17]. It is likely that all the above-mentioned mechanisms as well as more enriched miRNA regulation might increasingly enhance the tunability of these genes with low-copy number. As a result, their expression diversity and regulatory robustness could increase accordingly. This observation may imply that the characteristics of newly generated regulatory systems may randomly occur, but some with these characteristics may be maintained by natural selection during the evolutionary processes.

We fascinatingly found that duplication-generated miRNA genes in particular prefer to regulate genes in segmental duplication blocks, whereas they failed to preferably regulate genes in tandem duplication arrays. This phenomenon may reflect a gene balance effect in the evolution of miRNA regulatory systems [49]. With the expansion of gene families generated by the whole genome duplication or segmental duplication events, physically connected miRNA families also increased in the same manner as coding genes to maintain a stoichiometric balance of genes involved in these regulatory systems. However, the disagreement appears that, on one hand, given that target gene families expanded when gene duplications occur, the target number of these miRNAs should be relatively large. On the other hand, our observations together with previous studies [50] showed that target gene number of these miRNAs was relatively small. The most likely explanation is that, miRNA target sites may be lost or diverged in the processes of the neofunctionalization and subfunctionalization of these duplicate genes, as the divergence of their regulations commonly occur [51], [52].

It is our belief that the origin of miRNA genes may independently occur in animal and plant kingdom [53]. The small RNAs which have been detected in animals are mainly miRNAs [3]. Contrastingly, flowering plants possess relatively large number of small RNAs, but miRNAs only occupy a minority of the whole small RNA set in the genome [3]. Although this study cannot provide the evidence that miRNAs are able to directly furnish genetic robustness in flowering plants, these miRNAs could predominantly offer the robustness for gene regulatory system through regulatory buffering. Such a regulatory buffering is highly congruent and might indirectly supply genetic robustness. By contrast, miRNAs tend to regulate genes with high-copy number and thus are able to directly provide genetic robustness in animals [54]. Indeed, it is possible that genetic robustness could also be provided by other small RNAs, such as siRNAs in the genome. Therefore, efforts are looked-for to gain deep insights into other long-standing issues regarding the origins and evolution of siRNAs in flowering plants.

## Materials and Methods

### Data source

miRNA sequences of *O. sativa* and *A. thaliana* were downloaded from the release 13.0 of the miRBase database (http://microrna.sanger.ac.uk/). Although miRBase has extensively been accepted as a standard for miRNA annotation, some released miRNAs might be incorrectly annotated and not assessed with sufficient stringent criteria prior to their addition to the database. To eliminate these potentially non-authentic miRNAs, we applied the MIRcheck program (http://web.wi.mit.edu/bartel/pub/software.html) to select miRNAs which have ≤4 mismatches, ≤2 continuous mismatches in both miRNA and putative miRNA* sequences, ≤2 asymmetrically unpaired nucleotides in miRNA sequences [3]. Of 357 and 187 miRNA genes in *O. sativa* and *A. thaliana* genomes, 290 and 142 satisfied this criteria. Genome sequences, annotation data as well as alternative splicing data of the *O. sativa* ssp. *japonica, O. sativa* ssp. *indica* and *A. thaliana* were collected from the release 6.0 of The Institute of Genome Research (TIGR) database (ftp://ftp.tigr.org), Beijing Genomics Institute (BGI) Rice Information System (http://rice.genomics.org.cn/rice/index2.jsp) and the release 8 of The *Arabidopsis* Information Resources (TAIR) database (http://www.arabidopsis.org/), respectively. All TE-related genes and non-coding genes were excluded in the further analysis. For genes with alternative splice forms, we merely selected the transcripts with the longest sequences in length. Binding sites of miRNAs on protein-coding genes of *O. sativa* and *A. thaliana* were identified by using psRNATarget server (http://bioinfo3.noble.org.psRNATarget/) with default parameters [55]. psRNATarget server is an update version of miRU server, which has been a popular tool for plant miRNA target analysis. Pseudogene data of *O. sativa* and *A. thaliana* were taken from the Pseudogene.org database (http://www.pseudogene.org/databases.html).

### Identification of miRNA genes from different origin modes

Repetitive sequences, including TEs and simple sequence repeats (SSRs) in both *O. sativa* and *A. thaliana* genomes, were identified by using the RepeatMasker program (http://www.repeatmasker.org) with default parameters. miRNA genes overlapped with TEs or with at least 50% coverage with TEs were regarded as TE-related miRNA gene. Similarly, miRNA genes overlapped with pseudogenes or with at least 50% coverage with pseudogenes were considered as pseudogene-related miRNA gene. Inverted duplication-generated miRNA genes were identified by using the approaches as below: (1) pre-miRNA sequences were subject to FASTA searches against cDNA sequences; (2) cases that reverse complemented pre-miRNA sequence matched with cDNA sequence with E-value<0.05, identity-value>0.8 and miRNA sequence lies in the matched regions were selected. Tandem duplication generated miRNA genes were identified in quest of contiguous miRNA genes. In this study, we only included the pair of miRNA genes with a distance <15000 bp within the same family or their BLASTN E-value of pre-miRNA <e-10. Segmental duplications which generated miRNA gene pairs were identified by using DAGchainer program (ftp://ftp.tigr.org/pub/software/DAGchainer/) with parameters “-s –l”. They primarily include self-comparisons to ignore tandem duplication alignments. We used protein pairs with BLASTP E-value<e-10 and miRNA gene pairs within the same family or their BLASTN E-value of pre-miRNA <e-10 as the input data when running DAGchainer program.

### Characterization of features of miRNA genes and their target genes

Gene Ontology (GO) enrichment analyses of miRNA target genes were implemented by using the software GOEAST (http://omiclab.genetics.ac.cn/GOEAST/). Pre-miRNA secondary structures were predicted by using Vienna RNA Package (http://rna.tbi.univie.ac.at/). Promoters (TATA box) and enhancers of miRNA genes were identified from upstream 1 kb regions of pre-miRNAs by using the software TSSP (http://linux1.softberry.com/berry.phtml). siRNA sequences of *O. sativa* and *A. thaliana* were taken from the Cereal small RNA database (CSRDB, http://sundarlab.ucdavis.edu/smrnas/) and the *Arabidopsis* Small RNA Project (ASRP) database (http://asrp.cgrb.oregonstate.edu/), respectively. When we calculated siRNAs encoding miRNA precursors [14], [56], siRNAs overlapped with mature miRNAs were discarded for the subsequent data analysis. Here, we evaluated the conservation of miRNA genes across species by directly counting and collecting miRNA gene orthologs in a total of 14 land plant species in miRbase. These species are *O. sativa*, *A. thaliana*, *Triticum aestivum*, *Zea mays*, *Brassica napus*, *Medicago truncatula*, *Poplar trichocarpa*, *Vitis vinifera*, *Monodelphis domaestica*, *Glycine max*, *Selaginella moellendorffii*, *Pinus taeda*, *Physcomitrella patens* and *Sorghum bicolor*. miRNA expression data of *A. thaliana*, comprising of three tissues by 454 sequencing, were downloaded from ASRP database. For each miRNA, the mean of these three values were estimated and used. DNA methylation data of *A. thaliana* were downloaded from Annoj (http://neomorph.salk.edu/epigenome.html). We adopted data of polymorphic regions in *A. thaliana*, which composed of 20 datasets with resequencing microarrays [57].

To obtain detailed information of gene copy number, the protein sequence of each query gene was used to search against all other proteins using BLASTP (E<e-10). For proteins belonging to the same family, we used two criteria as below [58]: (1) their similarity is ≥I (I = 30% if L≥150 a.a. and I = 0.01n+4.8L^−0.32(1+exp)−L/1000))^ if L<150 a.a., where n = 6 and L is the length of the alignable region) and (2) the length of alignable region between the two sequences is ≥80% of the longer protein. Gene expression data from 40 cell types of *O. sativa* were downloaded from Yale Virtual Center for Cellular Expression Profiling of Rice (http://bioinformatics.med.yale.edu/riceatlas/download.jspx). Meanwhile, For *A. thaliana*, we obtained gene expression data from 79 tissues at Weigel world, (http://www.weigelworld.org/resources/resources/microarray/AtGenExpress).The mean of expression intensity was used as its expression level for each gene. For miRNAs with multiple target genes, however, average values of gene expression intensity, copy number and alternative splice number were collectively estimated. Segmental duplication blocks were simply identified by using DAGchainer with parameters the same as in the above-described analysis. Two paralogous genes were considered as tandem duplication when they are separated by fewer than 20 genes.

### Statistics analyses of relationships among different miRNA gene origin modes

To illustrate relationships among different miRNA gene origin modes, two methods were used here. First, random intersection number and statistical significance of each two datasets of miRNA genes with different origin modes were deduced by means of 100,000 Monte Carlo simulations. Briefly speaking, given M miRNA genes as the whole set of miRNA genes, assume that m of these M miRNA genes belong to dataset 1 and n of these M miRNA genes belong to dataset 2, and there are N miRNA genes belong to both of the two datasets. We randomly sampled m and n miRNA genes without replacement from the whole set of miRNA genes,respectively, and then counted intersection number of these two miRNA gene sets. We repeated the sampling in a number as large as 100,000 times in this study, and then took average intersection number as random one. The frequency of intersection number smaller than N was taken as an empirical *P*-value merely when such a number was larger than N. Otherwise, the frequency of intersection number larger than N was used as empirical *P*-value.

Second, we built discriminative models with the above-described features of miRNA genes to distinguish miRNA genes from their different origin modes implemented with Orange software (http://www.ailab.si/orange/). For each pair of miRNA gene datasets from different origin modes, six discriminative models were built by using six different classifiers, including Naive Bayes, k Nearest Neighbours, Decision Tree, C4.5, Support Vector Machine (SVM) and Random Forest. Once a discriminative model was built, it can be used to determine a miRNA gene's origin. The accuracy estimation was performed for the discriminative model by using 5-fold cross-validation method. Original dataset was randomly divided into five equally sized subsets and then in i-th iteration (i = 1..5) i-th subset was used for testing the prediction accuracy of the model that has been built on other remaining subsets. The average accuracy of these tests was the final accuracy of this model. The average accuracy of six discriminative models was the final accuracy of every two datasets.

## Supporting Information

Figure S1 **Flowchart of this study.**
(PDF)

Figure S2 **Schematic figure of osa-MIR806h.** (A) osa-MIR806h and flanking regions in the *japonica* genome and ortholous regions in the *indica* genome. Light blue boxes represent gene, red box represent MITE, and dash lines represent homologous relationships between genes in the *japonica* and *indica* genomes. (B) Predicted fold-back structure of osa-MIR806h precursor. Red line represents mature miRNA sequence.
(PDF)

Figure S3 **Schematic figure of osa-MIR446 and its target.** (A) osa-MIR446's target on LOC_Os08g05620 in the *japonica* genome and ortholous regions in the *indica* genome. (B) FASTA alignments of osa-MIR446 and LOC_Os08g05620. Red regions represent MITE, while blue regions represent mature miRNA sequence and its target sequence. (C) Predicted fold-back structure of osa-MIR446 precursors. Red line represents mature miRNA sequence.
(PDF)

Figure S4 **Phylogenetic analyses of MITEs overlapped with some miRNA genes and their targets.** (A) MITEs overlapped with osa-MIR806h, targets of osa-MIR806h and other osa-MIR806 family members. (B) MITEs overlapped with osa-MIR446, its target on LOC_Os08g05620 and other genes. Phylogenetic trees were built through Neighbor-Joining method by using ClustalW and Phylip with default parameters.
(PDF)

Table S1 Summary of miRNA genes with different modes of origin in *O. sativa* and *A. thaliana*.
(XLS)

Table S2 Statistics for TEs overlapped with miRNA genes, TEs overlapped with miRNA targets and TEs distributed in the *O. sativa* and *A. thaliana* genomes.
(DOC)

Table S3 Difference among miRNA genes with different modes of origin in *O. sativa* and *A. thaliana*.
(XLS)

Table S4 Relationships of miRNA genes with different origins.
(DOC)

Table S5 A list of 58 selected miRNA genes.
(XLS)

Table S6 Difference between miRNA genes overlapped with non-MITEs and MITEs in *O. sativa*.
(DOC)

Table S7 Summary of miRNA genes *de novo* generated accorded with model (C).
(XLS)

Table S8 Summary of miRNA genes *de novo* generated accorded with model (D).
(XLS)
