# Supplementary figures and images for: Evolution of MicroRNA Genes in Oryza sativa and Arabidopsis thaliana: An Update of the Inverted Duplication Model
Source: PLoS One. 2011 Dec 14;6(12):e28073. doi: 10.1371/journal.pone.0028073 (PMC3237417; doi:10.1371/journal.pone.0028073)

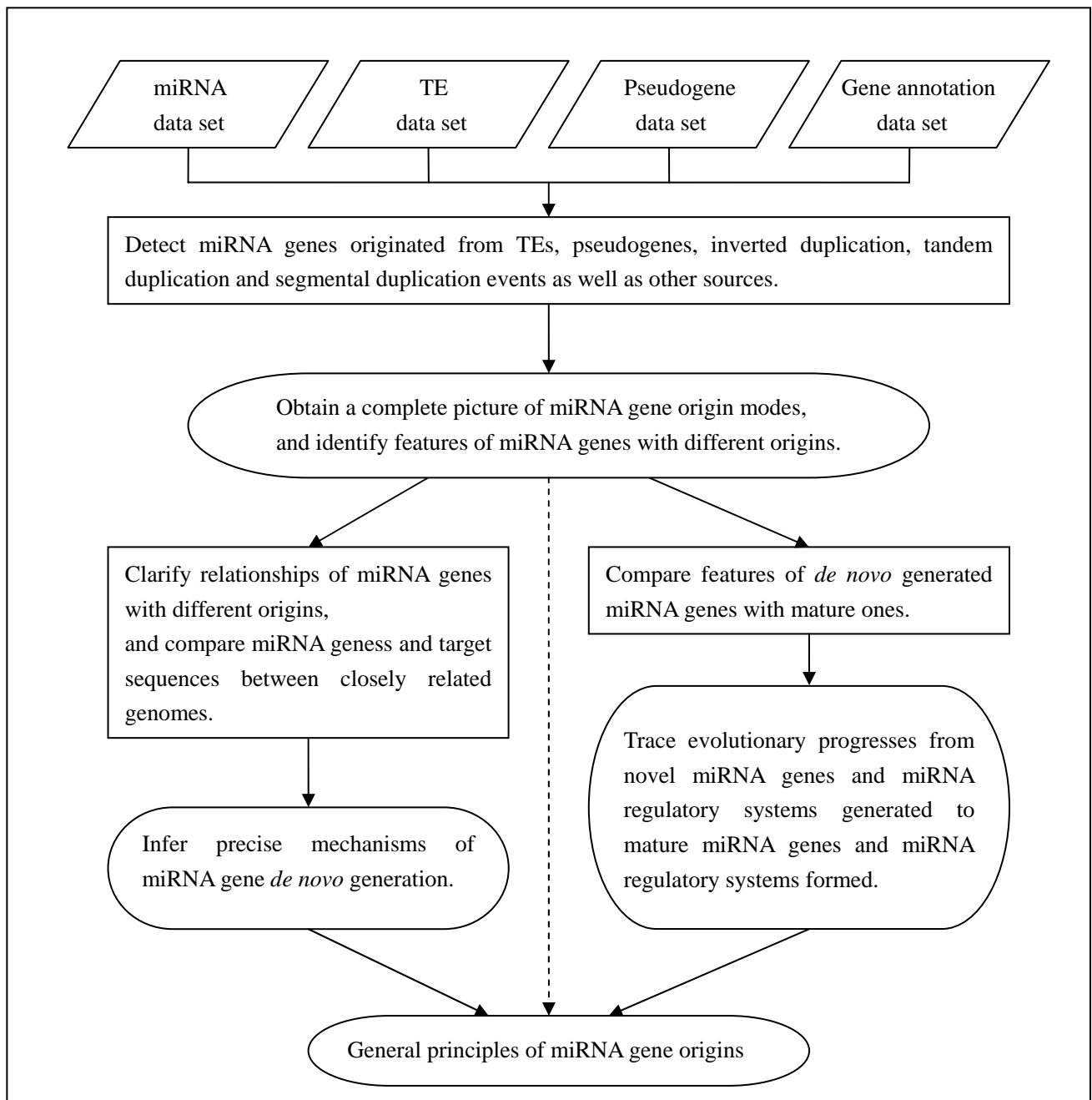

Supplement: Figure S1 — Flowchart of this study. (PDF) [file pone.0028073.s001.pdf]

**A**

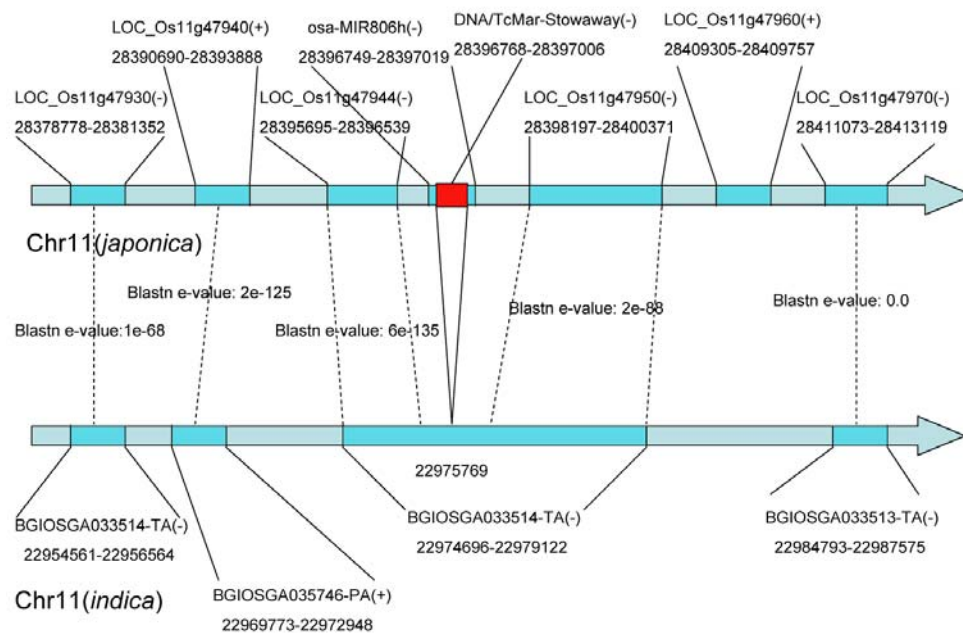

**B**

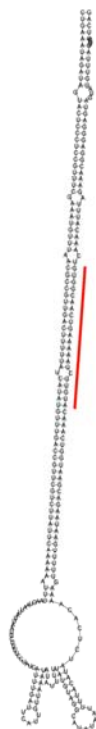

Supplement: Figure S2 — Schematic figure of osa-MIR806h. (A) osa-MIR806h and flanking regions in the japonica genome and ortholous regions in the indica genome. Light blue boxes represent gene, red box represent MITE, and dash lines represent homologous relationships between genes in the japonica and indica genomes. (B) Predicted fold-back structure of osa-MIR806h precursor. Red line represents mature miRNA sequence. (PDF) [file pone.0028073.s002.pdf]

**A**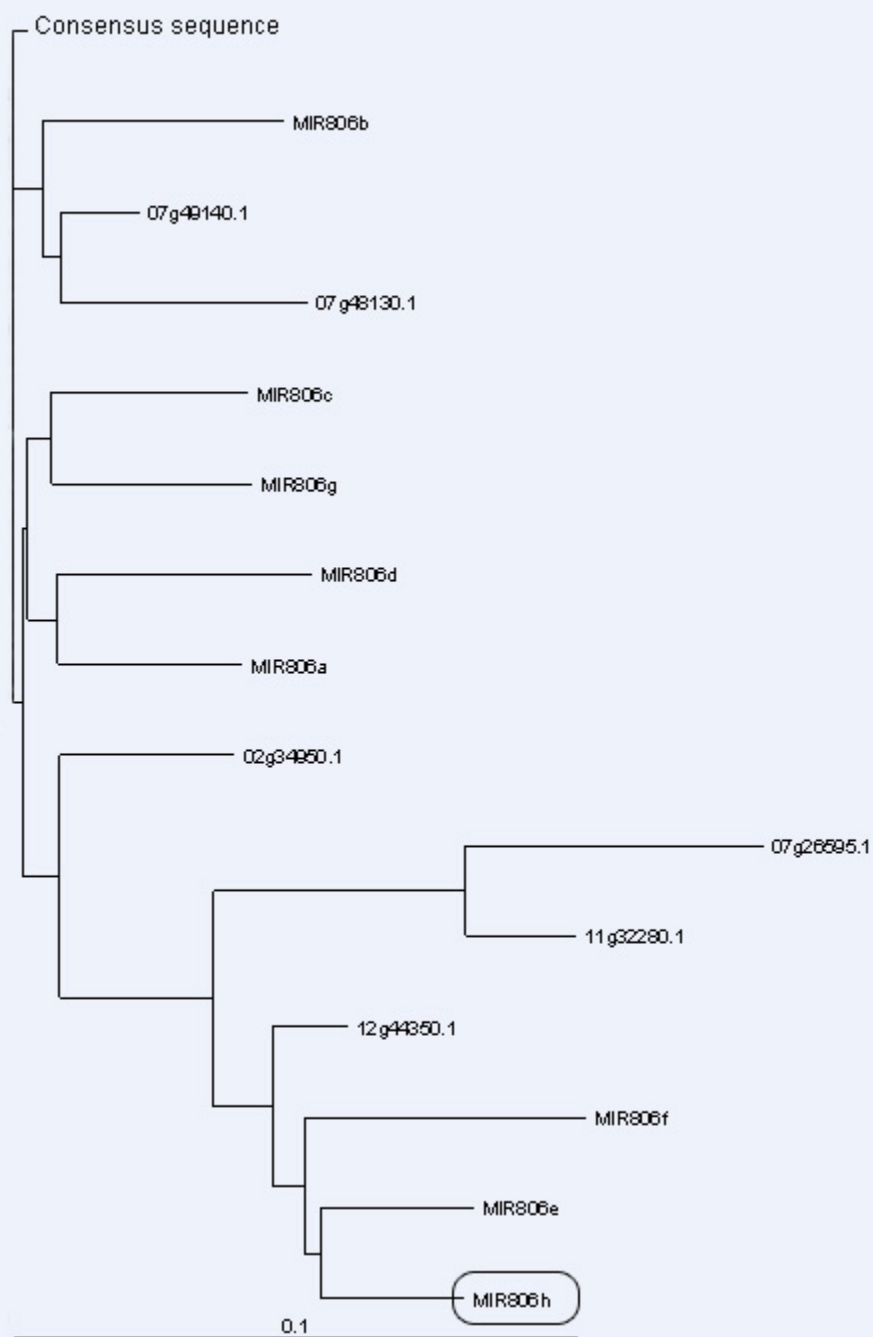**B**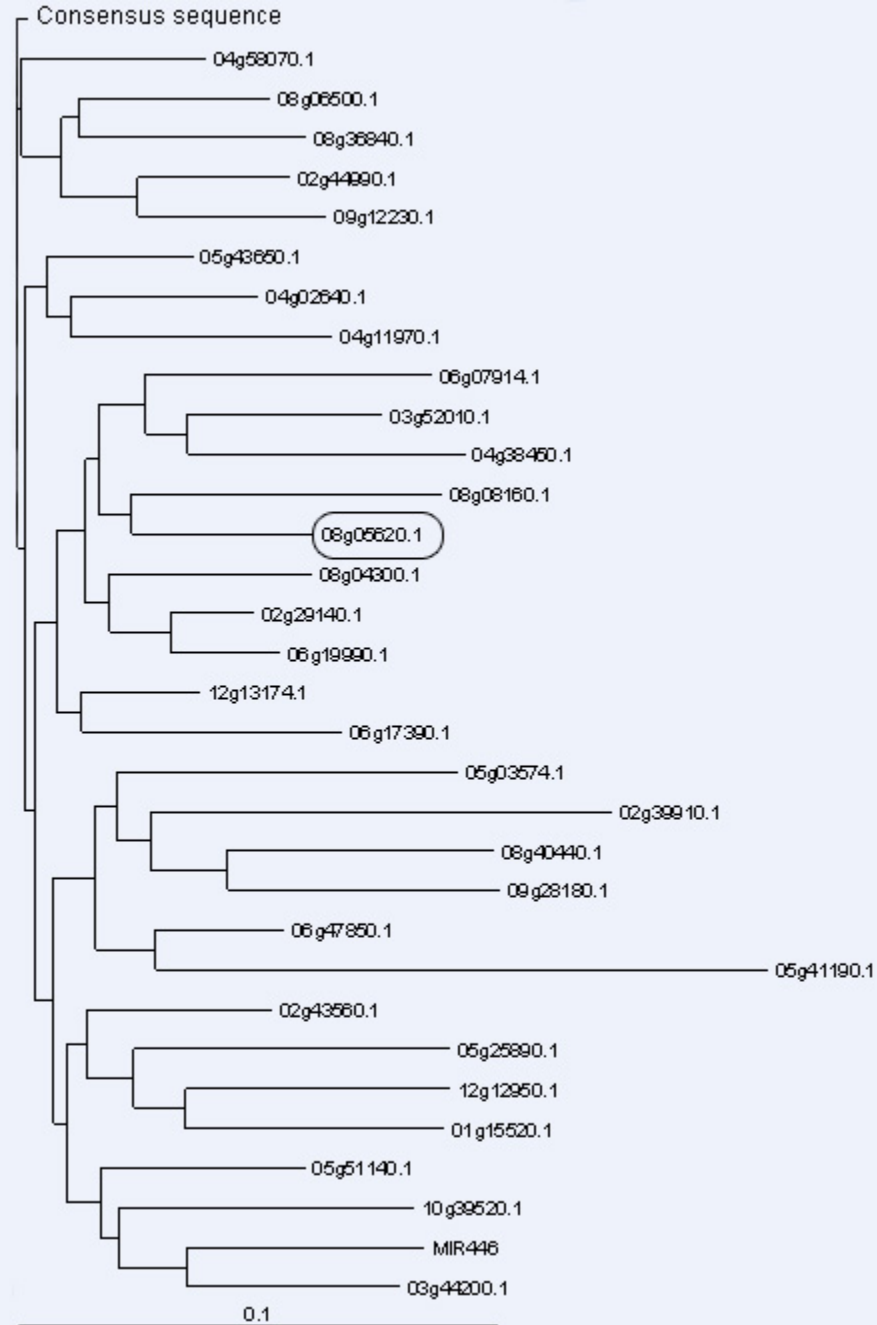

Supplement: Figure S4 — Phylogenetic analyses of MITEs overlapped with some miRNA genes and their targets. (A) MITEs overlapped with osa-MIR806h, targets of osa-MIR806h and other osa-MIR806 family members. (B) MITEs overlapped with osa-MIR446, its target on LOC_Os08g05620 and other genes. Phylogenetic trees were built through Neighbor-Joining method by using ClustalW and Phylip with default parameters. (PDF) [file pone.0028073.s004.pdf]
