# Supplementary material for: Evolution of MicroRNA Genes in Oryza sativa and Arabidopsis thaliana: An Update of the Inverted Duplication Model
Source: PLoS One. 2011 Dec 14;6(12):e28073. doi: 10.1371/journal.pone.0028073 (PMC3237417; doi:10.1371/journal.pone.0028073)

A

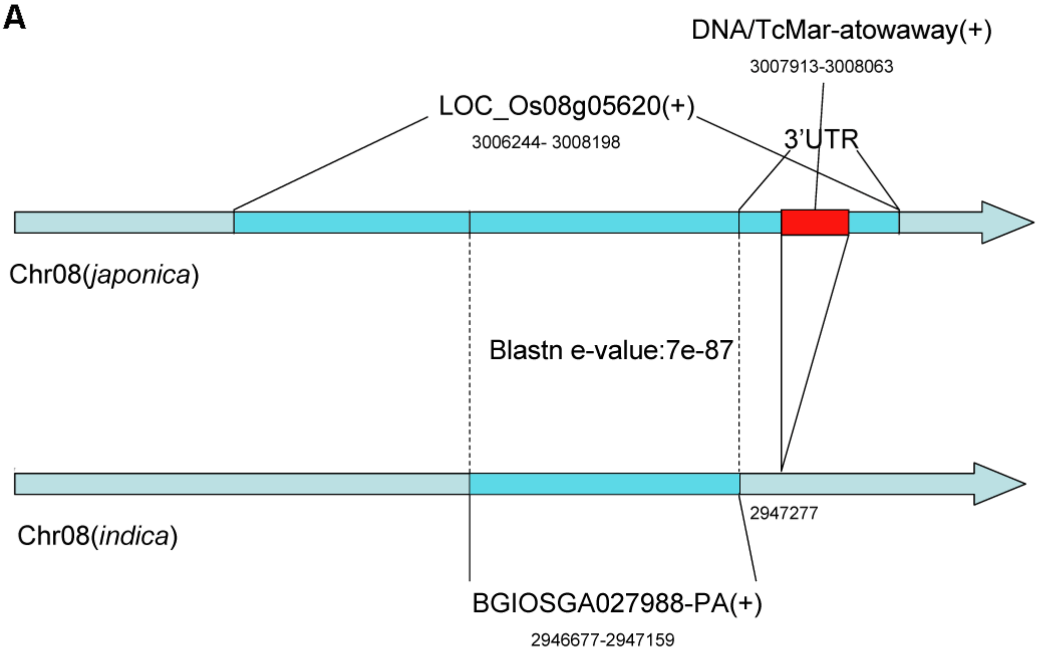

B

```
>LOC_Os08g05620.1|13108.m00554|cDNA cytochrome P450, pu  (1955 nt)
rev-comp initn: 560 init1: 560 opt: 603  Z-score: 450.1  bits: 93.3 E(): 8.4e-19
 85.119% identity (85.629% ungapped) in 168 nt overlap (168-2:1661-1828)

                              170       160       150       140
osa-M-                        GTTCAAGTTCTATATACTCCCTCCGTTTCACAATGTA
                                     : :::  :::: ::::::::: ::::::::
LOC_Os TAGGTCATGGCTTCTCTTTGCTAATCATGCTACTAATTACTTCCTCCGTTTTACAATGTA
            1640      1650      1660      1670      1680      1690

            130       120       110       100        90        80
osa-M- AGTTATTCTACCATTTCCCATATTCATATTGATGTTAATAAATCTAGACATATATATCTA
       ::: :::::  :::::: ::::::::::::::: :::  :::::::::::::::::::
LOC_Os AGTCATTCTGGCATTTCTCATATTCATATTGATGCTAACGAATCTAGACATATATATCTA
            1700      1710      1720      1730      1740      1750

             70        60        50        40        30        20
osa-M- TATAGATTCATTAACATCAATATAAATGTGGGAAATTTTATAATGACTTACACTGTGAAA
       : :::::::::::::: ::::: ::: :::::::: ::: ::::::::::: :::::::
LOC_Os TCTAGATTCATTAACATTAATATGAATATGGGAAATGTTAGAATGACTTACATTGTGAAA
            1760      1770      1780      1790      1800      1810

              10
osa-M- C-AAAGGAAGTACATATTT
       :   ::::::::   :::
LOC_Os CTGGAGGAAGTAGCAATTAAAAATGGTGATTAGATTTAAGTTCCTCTTGTTCTTTTGGGG
            1820      1830      1840      1850      1860      1870
```

C

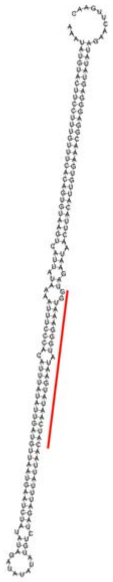

Supplement: Figure S3 — Schematic figure of osa-MIR446 and its target. (A) osa-MIR446's target on LOC_Os08g05620 in the japonica genome and ortholous regions in the indica genome. (B) FASTA alignments of osa-MIR446 and LOC_Os08g05620. Red regions represent MITE, while blue regions represent mature miRNA sequence and its target sequence. (C) Predicted fold-back structure of osa-MIR446 precursors. Red line represents mature miRNA sequence. (PDF) [file pone.0028073.s003.pdf]
