# Supplementary material for: Evolution of MicroRNA Genes in Oryza sativa and Arabidopsis thaliana: An Update of the Inverted Duplication Model
Source: PLoS One. 2011 Dec 14;6(12):e28073. doi: 10.1371/journal.pone.0028073 (PMC3237417; doi:10.1371/journal.pone.0028073)
Supplement: Table S2 — Statistics for TEs overlapped with miRNA genes, TEs overlapped with miRNA targets and TEs distributed in the O. sativa and A. thaliana genomes. (DOC) [file pone.0028073.s006.doc]

***Table S2a. TEs overlapped with miRNA genes in O. sativa genome.***

|  | *Number(a,b,c)** | *Percentage* | *Integrity* | *Divergence* | *Length* | *SW-value* |
| --- | --- | --- | --- | --- | --- | --- |
| **DNA** | 8(1,4,3) | 8.16% | 0.71±0.31 | 9.07±5.13 | 404.88±327.04 | 2497.13±1632.74 |
| **DNA/En-Spm** | 12(1,7,4) | 12.24% | 0.20±0.32 | 22.63±2.36 | 518.33±387.20 | 1559.42±1344.53 |
| **DNA/MuDR** | 4(1,1,2) | 4.08% | 0.29±0.48 | 22.72±6.50 | 634.50±615.94 | 1199.00±1048.05 |
| **DNA/Stowaway** | 5(3,2,0) | 5.10% | 0.66±0.29 | 18.14±3.57 | 95.60±41.70 | 368.60±138.97 |
| **DNA/TcMar-Stowaway** | 61(8,53,0) | 62.24% | 0.91±0.20 | 13.74±4.40 | 163.54±57.87 | 938.39±404.73 |
| **DNA/Tourist** | 6(0,6,0) | 6.12% | 0.85±0.24 | 13.18±5.32 | 304.17±82.93 | 1755.33±759.51 |
| **DNA/hAT** | 2(1,1,0) | 2.04% | 0.87±0.19 | 19.80±5.80 | 140.50±70.00 | 554.00±453.96 |
| **Total** | 98(15,74,9) | 100.00% | 0.77±0.35 | 15.13±5.74 |  |  |

*a: number of TE in length <= 100bp;

*b: number of TE in length > 100bp and <= 600bp;

*c: number of TE in length > 600bp.

** Most of TEs (74/98) overlapped with miRNA genes are short transposons (100-600bp).

***Table S2b. TEs overlapped with miRNA genes in A. thaliana genome.***

|  | *Number(a,b,c)** | *Percentage* | *Integrity* | *Divergence* | *Length* | *SW-value* |
| --- | --- | --- | --- | --- | --- | --- |
| **DNA/MuDR** | 2(0,2,0) | 40.00% | 0.03±0.00 | 18.10±0.28 | 121.50±9.19 | 623.00±5.66 |
| **DNA/hAT** | 3(0,3,0) | 60.00% | 0.33±0.02 | 10.27±0.91 | 400.67±21.57 | 2333.33±345.58 |
| **Total** | 5(0,5,0) | 100.00% | 0.21±0.16 | 13.40±4.34 |  |  |

*a: number of TE in length <= 100bp;

*b: number of TE in length > 100bp and <= 600bp;

*c: number of TE in length > 600bp.

**Table S2c. TEs overlapped with miRNA targets in *O. sativa* genome.**

|  | *Number(a,b,c)** | *Percentage* | *Integrity* | *Divergence*** | *Length* | *SW-value* |
| --- | --- | --- | --- | --- | --- | --- |
| **DNA** | 181(45,126,10) | 10.93% | 0.21±0.24 | 19.84±3.88 | 196.80±223.82 | 637.70±677.82 |
| **DNA/En-Spm** | 27(3,18,6) | 1.63% | 0.19±0.33 | 20.61±8.87 | 1500.93±3126.73 | 10527.96±24579.50 |
| **DNA/MuDR** | 25(5,12,8) | 1.51% | 0.23±0.36 | 16.48±12.00 | 1498.76±2756.16 | 12956.88±24932.71 |
| **DNA/Stowaway** | 170(52,118,0) | 10.27% | 0.76±0.27 | 13.40±4.60 | 109.20±35.35 | 548.41±262.60 |
| **DNA/TcMar-Stowaway** | 1195(186,1009,0) | 72.16% | 0.90±0.20 | 11.70±4.61 | 162.20±57.40 | 979.08±400.25 |
| **DNA/Tourist** | 45(0,45,0) | 2.72% | 0.95±0.12 | 7.27±5.43 | 322.27±52.59 | 2443.51±695.00 |
| **DNA/hAT** | 1(0,1,0) | 0.06% | 1.00±0.00 | 11.10±0.00 | 188.00±0.00 | 827.00±0.00 |
| **DNA/hAT-Tip100** | 1(0,0,1) | 0.06% | 0.41±0.00 | 33.60±0.00 | 1424.00±0.00 | 1063.00±0.00 |
| **LINE/L1** | 9(0,2,7) | 0.54% | 0.50±0.33 | 8.24±9.80 | 3463.22±2530.30 | 29490.00±24061.10 |
| **LTR/Copia** | 1(0,1,0) | 0.06% | 0.19±0.00 | 6.00±0.00 | 1290.00±0.00 | 8482.00±0.00 |
| **LTR/Gypsy** | 1(0,1,0) | 0.06% | 0.15±0.00 | 19.70±0.00 | 469.00±0.00 | 1885.00±0.00 |
| **Total** | 1656(291,1333,32) | 100.00% | 0.79±0.32 | 12.86±5.70 |  |  |

*a: number of TE in length <= 100bp;

*b: number of TE in length > 100bp and <= 600bp;

*c: number of TE in length > 600bp.

**Divergence = mismatches of consensus sequence/(matches+mismatches)

***Most of TEs (1333/1656) overlapped with miRNA targets are short transposons (100-600bp).

****Divergence of TEs overlapped with miRNA genes(15.13±5.74) is larger than that with miRNA targets(12.86±5.70, *t*-test *P*<0.000). These results indicate that average age of TEs overlapped with miRNA genes is older than those with miRNA targets.

***Table S2d. TEs overlapped with miRNA targets in A. thaliana genome.***

|  | *Number(a,b,c)** | *Percentage* | *Integrity* | *Divergence* | *Length* | *SW-value* |
| --- | --- | --- | --- | --- | --- | --- |
| **LTR/Gypsy** | 2(0,0,2) | 66.67% | 0.27±0.00 | 26.30±00.00 | 818.00±0.00 | 1418．00±00.00 |
| **RC/Helitron** | 1(0,1,0) | 33.33% | 0.19±0.00 | 17.00±0.00 | 397±0.00 | 742±0.00 |
| **Total** | 3(0,1,3) | 100.00% | 0.24±0.04 | 23.20±5.37 |  |  |

*a: number of TE in length <= 100bp;

*b: number of TE in length > 100bp and <= 600bp;

*c: number of TE in length > 600 bp.

**Divergence of TEs overlapped with miRNA genes(13.40±4.34) is smaller than that with miRNA targets(23.20±5.37, *t*-test *P*=0.062). These results indicate that average age of TEs overlapped with miRNA genes is younger than those with miRNA targets.

**Table S2e. TEs in *O. sativa* genome.**

|  | *Number(a,b,c)** | *Percentage* | *Integrity* | *Divergence* | *Length* | *SW-value* |
| --- | --- | --- | --- | --- | --- | --- |
| **DNA** | 60912(13140,45631,2141) | 20.12% | 0.43±0.34 | 18.51±6.38 | 207.44±172.38 | 780.41±692.32 |
| **DNA/En-Spm** | 23596(7022,12402,4172) | 7.79% | 0.15±0.24 | 18.89±7.75 | 595.01±1410.68 | 3060.88±8352.45 |
| **DNA/Harbinger** | 363(45,232,86) | 0.12% | 0.14±0.15 | 23.85±8.01 | 478.13±700.61 | 2200.24±6313.02 |
| **DNA/MuDR** | 15569(2613,11006,1950) | 5.14% | 0.28±0.30 | 19.94±8.69 | 425.80±932.28 | 2537.31±7742.34 |
| **DNA/MuDR?** | 796(133,662,1) | 0.26% | 0.51±0.33 | 20.51±7.20 | 210.74±126.15 | 874.10±868.97 |
| **DNA/Stowaway** | 9400(3906,5410,84) | 3.10% | 0.57±0.29 | 15.57±5.90 | 140.87±100.23 | 632.41±516.79 |
| **DNA/TcMar-Stowaway** | 43094(8147,34927,20) | 14.23% | 0.81±0.26 | 16.23±6.04 | 177.43±85.57 | 860.48±672.46 |
| **DNA/Tourist** | 42844(5861,36980,3) | 14.15% | 0.74±0.26 | 17.13±6.11 | 203.67±88.34 | 1008.96±686.78 |
| **DNA/hAT** | 5848(1126,4422,300) | 1.93% | 0.41±0.35 | 18.34±7.07 | 246.74±366.10 | 1261.19±3029.13 |
| **DNA/hAT-Ac** | 1204(329,782,93) | 0.40% | 0.16±0.27 | 20.85±7.86 | 248.37±469.93 | 1329.08±4426.40 |
| **DNA/hAT-Tip100** | 161(19,99,43) | 0.05% | 0.17±0.22 | 25.74±8.78 | 586.78±758.27 | 2090.71±5587.41 |
| **DNA/hAT?** | 475(118,357,0) | 0.16% | 0.56±0.35 | 17.49±7.49 | 221.9±159.48 | 1054.15±866.48 |
| **LINE/L1** | 6223(1451,3238,1534) | 2.06% | 0.12±0.18 | 23.95±8.09 | 542.75±835.73 | 1810.89±4574.94 |
| **LTR** | 3731(485,2234,1012) | 1.23% | 0.28±0.29 | 18.12±8.04 | 581.62±815.93 | 2710.71±4699.74 |
| **LTR/Copia** | 12594(1772,5710,5112) | 4.16% | 0.43±0.37 | 14.50±9.12 | 898.04±1228.68 | 5678.26±9582.98 |
| **LTR/Copia?** | 915(167,467,281) | 0.30% | 0.33±0.36 | 18.25±6.29 | 582.10±607.32 | 2660.92±3701.12 |
| **LTR/Gypsy** | 57860(4878,24434,28548) | 19.11% | 0.41±0.35 | 15.42±7.71 | 1135.05±1540.18 | 6678.73±10293.45 |
| **Other** | 522(125,397,0) | 0.17% | 0.74±0.13 | 14.95±4.86 | 107.82±29.40 | 701.70±189.24 |
| **Other/centromeric** | 3(0,2,1) | 0.00% | 0.54±0.24 | 34.00±3.52 | 715.67±317.22 | 932.33±4023.59 |
| **RC/Helitron** | 2052(816,1022,214) | 0.68% | 0.03±0.09 | 18.82±8.07 | 373.79±1087.84 | 1669.13±8458.11 |
| **SINE** | 9588(3860,5728,0) | 3.17% | 0.52±0.31 | 16.66±7.21 | 150.50±97.92 | 784.17±641.73 |
| **Satellite** | 562(80,419,63) | 0.19% | 0.49±0.28 | 20.63±4.88 | 530.61±2166.51 | 807.46±717.78 |
| **Unknown** | 4357(984,1983,1390) | 1.44% | 0.40±0.33 | 16.16±6.49 | 509.91±498.60 | 1850.00±2421.65 |
| **rRNA** | 130(40,70,20) | 0.04% | 0.15±0.26 | 21.51±8.20 | 372.96±673.74 | 1884.91±4754.29 |
| **Total** | 302799  (57117,198614,47068) | 100.00% | 0.49±0.37 | 17.28±7.28 |  |  |

*a: number of TE in length <= 100bp;

*b: number of TE in length > 100bp and <= 600bp;

*c: number of TE in length > 600 bp.

**Table S2f. TEs in *A. thaliana* genome.**

|  | *Number(a,b,c)** | *Percentage* | *Integrity* | *Divergence* | *Length* | *SW-value* |
| --- | --- | --- | --- | --- | --- | --- |
| **DNA** | 1338(175,1118,45) | 5.15% | 0.37±0.33 | 17.35±5.75 | 244.18±178.05 | 856.02±1164.48 |
| **DNA/En-Spm** | 843(98,381,364) | 3.25% | 0.15±0.22 | 19.57±9.25 | 1167.27±1702.96 | 5769.09±11292.22 |
| **DNA/Harbinger** | 368(60,202,106) | 1.42% | 0.22±0.30 | 20.16±8.58 | 471.90±546.93 | 1882.89±3919.95 |
| **DNA/Mariner** | 29(9,20,0) | 0.11% | 0.62±0.27 | 14.55±4.14 | 148.28±61.46 | 592.10±291.98 |
| **DNA/MuDR** | 3991(646,2281,1064) | 15.38% | 0.21±0.28 | 17.00±8.63 | 760.91±1632.66 | 4163.29±11075.15 |
| **DNA/Pogo** | 219(40,164,15) | 0.84% | 0.46±0.32 | 13.94±4.96 | 266.75±184.11 | 1073.18±979.68 |
| **DNA/Tc1** | 17(5,12,0) | 0.07% | 0.58±0.30 | 15.81±5.01 | 141.59±68.56 | 512.47±295.79 |
| **DNA/TcMar-Mariner** | 31(1,30,0) | 0.12% | 0.91±0.18 | 9.75±7.25 | 215.94±41.75 | 1341.61±499.03 |
| **DNA/TcMar-Pogo** | 283(67,197,19) | 1.09% | 0.48±0.37 | 15.04±5.67 | 291.69±227.38 | 1479.18±1948.54 |
| **DNA/TcMar-Stowaway** | 300(32,268,0) | 1.16% | 0.79±0.25 | 15.12±4.73 | 205.54±66.47 | 896.02±476.37 |
| **DNA/hAT** | 1116(207,720,189) | 4.30% | 0.40±0.35 | 16.49±8.67 | 368.19±430.77 | 1974.95±3155.56 |
| **DNA/hAT-Ac** | 130(30,81,19) | 0.50% | 0.19±0.23 | 20.34±8.70 | 379.93±575.35 | 1853.24±4203.80 |
| **DNA?** | 113(32,80,1) | 0.44% | 0.49±0.30 | 19.62±9.43 | 175.86±108.97 | 892.62±868.74 |
| **LINE/L1** | 1717(361,849,507) | 6.62% | 0.21±0.27 | 25.30±8.26 | 700.04±1064.29 | 2133.62±5798.20 |
| **LINE?** | 76(15,54,7) | 0.29% | 0.23±0.20 | 21.76±5.90 | 273.04±243.24 | 976.04±1426.99 |
| **LTR** | 735(582,153,0) | 2.83% | 0.32±0.29 | 17.42±5.12 | 102.32±88.88 | 566.29±529.75 |
| **LTR/Copia** | 2284(414,1307,563) | 8.80% | 0.45±0.41 | 15.62±10.67 | 748.37±1230.24 | 4619.51±9754.92 |
| **LTR/Gypsy** | 5881(666,2221,2994) | 22.66% | 0.38±0.36 | 13.76±8.94 | 1041.68±1295.02 | 5983.49±8338.42 |
| **Other/Composite** | 179(33,124,22) | 0.69% | 0.16±0.15 | 18.00±6.58 | 333.62±339.37 | 1596.07±2310.41 |
| **Other/centromeric** | 6(2,3,1) | 0.02% | 0.39±0.31 | 14.92±7.41 | 320.83±319.64 | 1927.83±2284.34 |
| **RC/Helitron** | 4109(818,2280,1011) | 15.83% | 0.31±0.34 | 13.03±7.55 | 512.15±810.89 | 2773.16±5629.87 |
| **SINE** | 624(173,432,19) | 2.40% | 0.66±0.31 | 17.00±8.12 | 174.63±142.84 | 1036.50±1242.64 |
| **SINE?** | 19(4,13,2) | 0.07% | 0.21±0.24 | 17.44±8.45 | 287.42±337.70 | 1739.11±2864.30 |
| **Satellite** | 730(331,333,66) | 2.81% | 0.60±0.29 | 19.56±6.86 | 797.00±4455.30 | 726.40±1543.52 |
| **Satellite/centr** | 793(115,584,94) | 3.06% | 0.89±0.22 | 23.02±3.91 | 586.04±1689.99 | 731.92±233.48 |
| **rRNA** | 25(15,6,4) | 0.10% | 0.19±0.36 | 11.93±8.95 | 499.60±1004.74 | 4247.00±9032.83 |
| **Total** | 25956  (4931,13913,7112) | 100.00% | 0.36±0.36 | 16.42±8.92 |  |  |

*a: number of TE in length <= 100bp;

*b: number of TE in length > 100bp and <= 600bp;

*c: number of TE in length > 600 bp.
