# Supplementary material for: Evolution of MicroRNA Genes in Oryza sativa and Arabidopsis thaliana: An Update of the Inverted Duplication Model
Source: PLoS One. 2011 Dec 14;6(12):e28073. doi: 10.1371/journal.pone.0028073 (PMC3237417; doi:10.1371/journal.pone.0028073)
Supplement: Table S4 — Relationships of miRNA genes with different origins. (DOC) [file pone.0028073.s008.doc]

|  | *TE*  *-related*  *(85) ^a^* | *Pseudogene*  *-related*  *(3)* | *Inverted duplication*  *(93)* | *Tandem duplication*  *(56)* | *Segmental duplication*  *(55)* |
| --- | --- | --- | --- | --- | --- |
| miRNA genes which targets overlapped with TEs(112) | 73 ^b^(32.94,  *P*=0.0000) ^c^ | 2 (1.17,  *P*=0.3344) | 78(36.05,  *P*=0.0000) | 1(21.70,  *P*=0.0000) | 3(21.31,  *P*=0.0000) |
| TE-related miRNA genes which targets overlapped with same TE(63) | 63(18.54,  *P*=0.0000) | 1(0.66,  *P*=0.5244) | 54(20.28,  *P*=0.0000) | 0(12.20,  *P*=0.0000) | 0(11.99,  *P*=0.0000) |

**Table S4a.** Intersection number of miRNA gene datasets with different origins in *O. sativa.*

^a^ miRNA number of this dataset.

^b^ Real value.

^c^ Random value and *P*-value in parentheses were deduced from 100,000 Monte Carlo simulation.

|  | *TE*  *-related*  *(4) ^a^* | *Pseudogene*  *-related*  *(4)* | *Inverted duplication*  *(11)* | *Tandem duplication*  *（22)* | *Segmental duplication*  *(46)* |
| --- | --- | --- | --- | --- | --- |
| miRNA genes which targets overlapped with TEs(3) | 1 ^b^(0.08,  *P*=0.0845)^c^ | 0 (0.08,  *P*=0.0000) | 0(0.23,  *P*=0.0000) | 0(0.47,  *P*=0.0000) | 0(0.98,  *P*=.0.0000) |
| TE-related miRNA genes which targets overlapped with same TE(0) | / | / | / | / | / |

**Table S4b.** Intersection number of miRNA gene datasets with different origins in *A. thaliana.*

^a^ miRNA number of this dataset.

^b^ Real value.

^c^ Random value and P-value in parentheses were deduced from 100,000 Monte Carlo simulation.

**Table S4c.** Classification accuracy of miRNA gene datasets with different origins in *O. sativa*.

|  | *TE*  *-related* | *Pseudogene -related* | *Inverted duplication* | *Tandem duplication* | *Segmental duplication* |
| --- | --- | --- | --- | --- | --- |
| miRNA genes which targets overlapped with TEs | 69.0% | 95.5% | 73.2% | 88.1% | 80.5% |
| TE-related miRNA genes which targets overlapped with same TEs | 62.8% | 96.1% | 43.9% | 89.8% | 93.3% |

**Table S4d.** Classification accuracy of miRNA gene datasets with different origins in *A. thaliana*.

|  | *TE*  *-related* | *Pseudogene -related* | *Inverted duplication* | *Tandem duplication* | *Segmental duplication* |
| --- | --- | --- | --- | --- | --- |
| miRNA genes which targets overlapped with TEs | 65.0% | 73.3% | 77.2% | 86.7% | 91.9% |
| TE-related miRNA genes which targets overlapped with same TEs | / | / | / | / | / |
