# Supplementary material for: Evolution of MicroRNA Genes in Oryza sativa and Arabidopsis thaliana: An Update of the Inverted Duplication Model
Source: PLoS One. 2011 Dec 14;6(12):e28073. doi: 10.1371/journal.pone.0028073 (PMC3237417; doi:10.1371/journal.pone.0028073)
Supplement: Table S6 — Difference between miRNA genes overlapped with non-MITEs and MITEs in O. sativa. (DOC) [file pone.0028073.s010.doc]

**Table S6. Difference between miRNA genes overlapped with non-MITE and MITE in *O. sativa*.**

|  | *Non-MITE related* | *MITE related* | P*-value^a^* |
| --- | --- | --- | --- |
| First nucleotide(U,A) | 50.00%,50.00% | 36.92%,40.00% | 0.297,0.429 |
| miRNA length(nt) | 23.00±1.45 | 22.22±1.01 | 0.008 |
| Precursor length(nt) | 157.75±66.18 | 173.60±52.80 | 0.336 |
| Stem length(nt) | 76.98±33.43 | 84.36±26.53 | 0.357 |
| Loop length(nt) | 5.80±3.04 | 6.34±2.53 | 0.478 |
| Distance between miR and loop | 36.10±30.39 | 43.62±26.50 | 0.328 |
| Match ratio of hairpin | 80.36% | 80.57% | 0.921 |
| siRNA(#) | 0.90±1.62 | 1.80±2.76 | 0.171 |
| Multi-miRNA | 10% | 0% | 0.009 |
| Promoter(#) | 0.80 | 0.51 | / |
| SSR(#) | 0.55±0.95 | 0.46±0.79 | 0.707 |
| Location | 50% | 37% | 0.321 |
| Family size(#) | 1.80±0.95 | 5.94±3.26 | 0.000 |
| Conservation | 1.10±0.45 | 1.00±0.00 | 0.071 |
| Target number(#) | 5.15±5.07 | 26.69±26.57 | 0.001 |
| Base pairings of cleaving site | 1.67±0.28 | 1.60±0.35 | 0.343 |
| Target expression level | 332.72±342.61 | 362.75±220.85 | 0.715 |
| Target gene copy numer(#) | 3.38±2.84 | 4.78±3.38 | 0.074 |
| Target gene splice number(#) | 2.06±1.86 | 1.66±0.59 | 0.401 |
| Segmental duplication target gene | 13.77% | 30.31% | 0.001 |
| Tandem duplication target gene | 16.79% | 13.84 | 0.573 |

^a^ *t*-test

see Supplementary Table 3, for more details.
